# A Little Peek May Be Enough: How Small Hive Beetle Estimates Can Help Address Immediate Colony Management Needs

**DOI:** 10.3390/insects16050517

**Published:** 2025-05-13

**Authors:** Ethel M. Villalobos, Luis Medina Medina, Zhening Zhang, Scott Nikaido, Emanuel Miranda, Jason Wong, Jessika Santamaria, Micaela Buteler

**Affiliations:** 1 Department of Plant and Environmental Protection Sciences, University of Hawaii at Manoa, Honolulu, HI 96822, USA; zhening@hawaii.edu (Z.Z.); snikaido@hawaii.edu (S.N.); jsantama@hawaii.edu (J.S.); 2 Departamento de Apicultura Tropical, Campus Ciencias Biológicas y Agropecuarias, Universidad Autónoma de Yucatán, Mérida 97315, Mexico; 3 Centro de Investigaciones Apícolas Tropicales (CINAT), Universidad Nacional de Costa Rica, Heredia 40104, Costa Rica; emiranda@coronadorada.com; 4 Hawai’i State Department of Health, Ka ‘Oihana Olakino, Lanakila Avenue, Honolulu, HI 96817, USA; jason.wong@doh.hawaii.gov; 5 Instituto de Investigaciones en Biodiversidad y Medio Ambiente (CONICET-Universidad Nacional Del Comahue), Bariloche 8400, Río Negro, Argentina; micabuteler@comahue-conicet.gob.ar

**Keywords:** small hive beetle, *Aethina tumida*, detection, inspections, in-hive distribution, management, Africanized honeybees, European honeybees

## Abstract

Due to the ongoing global spread of the small hive beetle (SHB), *Aethina tumida*, there is a significant need for detection and practical management strategies against this pest. The standard inspection strategies for SHBs involve (1) detailed visual examination of the colony, which is challenging in areas with defensive bees, or (2) sampling beetles via traps, which requires repeated visits to the apiary and can be difficult for beekeepers with apiaries in rural areas. In this study, we modified the inspection sequence to examine the in-hive distribution of the beetle and assess whether a limited, yet targeted, inspection could provide valuable information on beetle infestation. We conducted our modified sampling in three different countries: Hawai’i (USA), Mexico, and Costa Rica. We found that targeted screening of the top areas of the hive (cover and top-side frames) provided reliable information about the relative prevalence of SHBs in a colony. The results also suggested that SHBs do not naturally congregate on a bare bottom board but migrate downward during inspection. Trap placement on the bottom floor of the hive may underestimate beetle presence in low to medium pest levels. The proposed inspection protocol is not influenced by the genetic origin of the bees (Africanized or European) and could be a practical alternative for assessing SHB infestation levels in honeybee colonies.

## Simple Summary

The small hive beetle (SHB; *Aethina tumida*) is an invasive nest parasite of honeybees that continues to spread to new regions. Surveillance is crucial to detecting new invasions and to implementing management strategies. Based on a visual inspection of the interior of the hives, we found that SHBs do not naturally congregate on the bottom board but migrate downward during the inspection. Our results showed that the sum of SHB adults captured on a hive’s cover and outermost frames of the top box was significantly correlated with the overall number of beetles captured per colony. The bees’ genetic origin, Africanized or European, did not appear to impact the distribution of the beetles within the hive. The inspection protocol proposed could be an alternative method to assess the relative SHB infestation in colonies and provide guidance on immediate management needs.

## 1. Introduction

The small hive beetle (SHB), *Aethina tumida* Murray 1867 (*Coleoptera: Nitidulidae*), a native insect of South Africa, has greatly expanded its geographical distribution since the first SHB adults were collected in 1996 in South Carolina, USA [1]. The dispersion of the SHB has been likely accelerated by the anthropogenic movement of colonies and hive materials [2,3,4]. Within the last 20 years, the beetle has been reported in new areas, including Hawai’i [5], the Philippines [6], Korea [7], Australia [8], China [9], two provinces in Italy, Sicily and Calabria [4,10,11,12], India [13], and in multiple countries across Latin America [14,15,16]. As is common with invasive species, the economic impact of the beetle in its native habitat is minimal. However, outside the endemic range, the beetle can have a significant impact as an apicultural pest [17].

In Hawai’i, the geographical isolation of the islands and the ban on live bee importation protected the state from the SHB for over 15 years since its discovery in the mainland US. However, this pest was first reported on the island of Hawai’i in 2010 [5], and despite already existing regulations against the movement of live bees between the islands, it spread to the five main islands in the archipelago within 5 years of detection. While all Hawaiian beekeepers were negatively affected by the SHB, its impact was amplified on Oahu and Hawai’i Island, where producers were learning to cope with the recent introduction of the “*Varroa destructor* mite” [18]. In Latin America, the SHB was first reported in Africanized honeybee colonies in Mexico in 2007 [19]. Since then, over a dozen countries in Central and South America have detected the arrival of this pest [15]. Based on official reports, the SHB’s dispersion across the Neotropics has not followed a continuous latitudinal spread from the US towards the southern ranges. Global SHB detections show a scattered chronological and geographical pattern across different countries, suggesting anthropogenic-assisted spread, isolated introductions to different regions [17,20,21], and/or reduced surveillance and reporting [15].

In response to the spread of the SHB, there is renewed interest in SHB detection strategies, monitoring protocols [22,23,24,25,26,27], and trap designs [24,28,29,30,31]. At the regional level, detection methods for SHB include the establishment of sentinel apiaries in risk areas [22,27,32,33,34] and the visual screening of colonies [35]. The use of SHB traps inside the hives has been promoted since the early 2000s and continues to be a standard detection and control method [36,37,38]. A few recent methods include the analysis of colony debris and honey to detect SHB DNA [26,38,39].

Sampling for this pest, however, remains complicated for several reasons: First, there is no established economic threshold for adult beetles/colonies; consequently, beetle levels are hard to interpret, especially when individual colonies vary in size and strength. Although it is suspected that high SHB density combined with a low bee population may result in colony collapse [40], there are conflicting reports on how, or if, the size of colonies is related to susceptibility to SHB infestation [41,42,43]. Second, there is no consensus on how the number of SHBs captured in the variety of traps available corresponds to total infestation in a colony. Although the number of adult beetles caught in bottom-board traps is believed to represent roughly 35% of all SHBs [44] in the colony, there is scant information for other types of traps. Third, accurate sampling based on detailed visual inspections [29] is extremely time-consuming and has been used mostly in small-sized colonies of European descent with a relatively mild temperament. Consequently, there is no information on how this kind of in-depth inspection would work in an apiary composed of Africanized honeybees (AHBs). Finally, there is evidence that beetle density may vary within different regions of the hive, which could impact the effectiveness of traps and overall inspection success [45,46].

This study presents data on the relative density of SHBs based on visual inspection of different areas of the hive: the cover, bottom board, center frames, and side frames. The study included colonies of European descent in Hawai’i and Africanized bee colonies in Mexico and Costa Rica. Based on the existing literature, and our own preliminary observations, we hypothesized that the in-hive SHB distribution may be confounded by the disturbance caused by the inspection. To examine the impact of a sequential top-to-bottom colony inspection, we altered the sequence and examined the bottom of the hive first and assessed the beetle distribution following this new sequence. We also examined whether the abundance of beetles in certain areas of the hive could be used as a proxy for the relative infestation of a colony. This study provides practical insights for tropical and subtropical regions where the SHB remains active year-round, and mixed-ancestry colonies pose sampling challenges. Our goal is to postulate an easy-to-use SHB sampling technique that can be incorporated into regular hive management procedures and that provides beekeepers with immediate information on the relative SHB infestation of their colonies.

## 2. Materials and Methods

### 2.1. Sampling Sites

The apiaries sampled are described below, including the total number of colonies examined at each site and their respective configurations (Table 1). Data were collected between December 2015 and February 2016 from 25 colonies at the Waimanalo Agricultural Research Station in Oahu, Hawai’i. In April 2024, six additional colonies were sampled at the same site. The colonies sampled were pure European honeybee stock; ten consisted of only one brood box, ten had one honey box (known colloquially as supers), eight had two supers, and three had three supers. Data on 15 Africanized colonies, all housed in single brood box colonies without honey supers, were collected from 23 January to 27 February 2019 in Merida, Mexico. The boxes were constructed locally, often from several wood pieces with irregularities and small cracks. Data on Africanized colonies from Costa Rica were collected from three apiaries. Two apiaries in Liberia, Guanacaste province, were sampled: six colonies on 6 November 2022, and nine colonies at another apiary on 5 April 2023. Five additional colonies were examined on 11 November 2022 in Santa Ana, San José province. Thirteen of the Costa Rican colonies had one super, and seven were a single deep. All colonies sampled in this study were housed in standard Langstroth hives, except for the Mexican colonies previously described.

### 2.2. Sampling Protocol

The inspection protocol used in this study aimed to minimize the initial disruptions associated with hive inspection, such as frame manipulation, light, and smoke, to examine the actual distribution of SHBs within the “hive at rest”. Typically, inspection protocols for the SHB recommend starting at the top of the hive, moving downward through the hive boxes, and ending with the inspection of the hive floor. We hypothesize that this type of inspection resulted in the movement of the beetles towards the bottom during the manipulation of the frames. Thus, as the first step, we conducted a visual inspection of the bottom board before opening the hive and examining the colony’s interior. The inspection protocol used in this study is as follows:

1. Inspection began by lifting the whole hive with the cover on and without smoking the entrance. Because the hive and the bottom board were not physically attached to any of the colonies we sampled, the hive’s floor was then exposed. Adult beetles found on the hive floor were captured, and the bottom board was then considered “SHB-free”. Beetles caught at this stage were assigned to BB, which stands for bottom board before inspection.
2. The hive was then placed back over the bottom board.
3. The within-colony inspection began by sampling the cover, which, depending on the site, could include the lid and an inner cover or just a cover. Covers were visually inspected and/or banged on a hard substrate to dislodge any SHB adults. When the cover is removed, elements that affect the beetle’s behavior (sunlight, frame movement, and smoke) begin to impact the results. Consequently, the cover inspection was performed quickly, and the observers began the inspection of the first set of side frames almost simultaneously to ensure that these two areas were as accurate a representation of the SHB distribution as possible.
4. The side frames (SFs), the two most lateral frames in a box, could belong to a honey box or a brood box, depending on the configuration of the colony. Beetles found on the side frames were removed by tapping the frames on a solid surface and collecting beetles directly from the frames. The SF of the box immediately under the cover was labeled Sf_T_, indicating that this was the “top” box of this colony. For colonies consisting of only a brood box, the brood box itself was considered the top box.
5. After the SFs were examined, a single frame from the center of each box was inspected for beetles. The center frame (Cf) could be from either a honey box or a brood box, depending on the hive configuration. The percent SHB infestation that is strictly based on a center frame from the brood box is labeled Cf_b_.

The inspection of the side frames always preceded the screening of the center frame for each box, and the frames were inspected while the box was in situ. If the colony was composed of multiple boxes, after each box inspection, the box was placed on top of the upside-down hive cover before proceeding to the next box. If the first box was a brood box (single deep colonies), the box was not lifted from the floor of the hive during the internal inspection.

1. After all boxes were inspected, the hive was reassembled, and the cover was placed back on. The observers looked for beetles that may have moved down to the cover during the manipulation of the hive, but none were found.
2. The final step of the inspection involved lifting the whole hive off the bottom board again and collecting all beetles found on the floor. Beetles caught on the floor of the hive at this stage were assigned to BA, which stands for bottom board after inspection.

In Hawai’i, data on SHB adults collected from the lid and the inner cover were presented jointly as “cover” for analysis. In Mexico, the colonies were not outfitted with inner covers; consequently, data were only collected on the underside of the lid or cover of the hive. In Costa Rica, the colonies had a cloth under the lid, so beetle counts included the cover and the cloth jointly as “cover”. In Hawai’i and Costa Rica, where beetle infestation during the study was low compared to Mexico, the examination and removal of SHBs from the top part, including the cover and Sf_T_, could be completed in about 3 to 5 min. However, because the protocol involves killing SHBs as encountered, the time required to complete the screening is influenced by the number of beetles found; thus, in Mexico, screening on the top areas of the hive took approximately 8 to 12 min.

### 2.3. Statistics

We used Kruskal–Wallis tests to compare the abundance and in-hive distribution of SHBs at the three sites: Hawaii, Mexico, and Costa Rica. Additionally, we utilized post hoc Dunn’s tests to examine pairwise comparisons between the sites when a Kruskal–Wallis test was significant. We compared the beetle abundance on the bottom floor before and after inspection for each site using Wilcoxon matched-pairs signed-rank tests.

To examine the impact of the high beetle infestation at the Mexico site on the beetle’s ability to invade the center brood frames, we examined the density of SHBs in the center frame between colonies with high infestation (above average) and low infestation (below average) using Mann–Whitney U-tests. Finally, we examined whether there was a correlation between the number of beetles found in different areas of the topmost box and the number of SHBs captured elsewhere in the hive using Pearson’s correlation tests for each variable. Therefore, we tested whether the number of beetles caught on the cover, the Sf_T_, the sum of cover and Sf_T_, and the Cf_T_, were correlated to the residual beetles (RBs) at the three study sites.

## 3. Results

### 3.1. SHB Abundance in Honeybee Colonies in Hawai’i, Mexico, and Costa Rica

In this study, 65 managed honeybee colonies were sampled, and 3015 SHB adults were captured. The total number of SHBs captured per colony differed significantly between the sites (Kruskal–Wallis, H = 33.91, *p* < 0.0001). In Hawai’i, where the bees are of European descent, the average capture per colony was 25.87 (SD = 18.59). The AHB colonies in Costa Rica had the lowest beetle levels, averaging 14.15 per hive (SD = 10.40). On the other hand, AHB colonies from Mexico yielded high SHB numbers, with an average of 137.9 (SD = 92.20) beetles per colony. A post hoc Dunn’s test also revealed that there were significant differences between all paired groups. HI and CR were the most similar of the pairs (*p* < 0.05, Z = 2.3); in comparison, the average SHB density in MX was much higher than the other two sites and the difference was highly significant (*p* < 0.0001 for both sets of paired comparisons, and Z values of 4.19 for the HI-MX pair and 5.77 for the CR-MX pair).

### 3.2. Presence of SHB Larvae in Colonies

There was no evidence of SHB larvae in the colonies from Hawai’i, Costa Rica, or Mexico during our study. This result was based on visually examining the frames and bottom boards before and after the hive inspection of both European honeybee (EHB) and Africanized honeybee (AHB) colonies.

### 3.3. Bottom Board Before and After Colony Inspection

**Figure 1:**
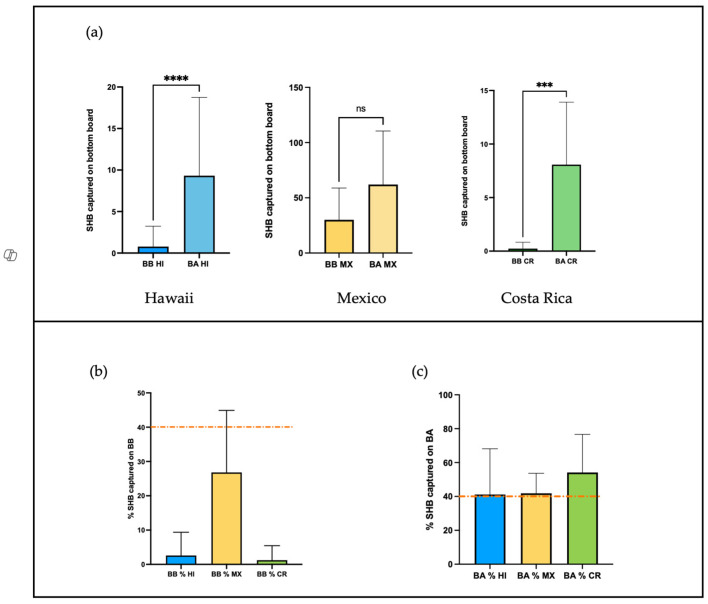
(**a**) The average number of SHBs caught on the bottom board before and after inspection at each site. Stars indicate the degree of significance between the before and after captures for each site. Non-significance is indicated by “ns”. The average percentage of SHBs found on the bottom board before (**b**) and after (**c**) inspection across sites. HI = Hawaii, MX = Mexico, and CR = Costa Rica. BB = bottom board before inspection and BA = bottom board after inspection. Whiskers indicate standard deviation. The dotted red line, arbitrarily set at 40%, is included as a visual aid to compare the prevalence of the SHBs on the bottom board at the three study sites.

We first present the results of the bottom board inspection for each site (Figure 1a). At each site, the total number of SHBs collected post-colony manipulation was higher than before the colony inspection began. However, a Wilcoxon matched-pairs signed-rank test showed that the SHB prevalence on the bottom board after inspection was significantly higher than before inspection for the two sites: HI *p* < 0.0001, W = 372 and CR *p* < 0.0001, W = 210. In Mexico, the difference in SHB numbers on the bottom board before and after inspection was below significance (*p* = 0.06).

Due to the large differences in beetle abundance between sites, we used the percentage of SHBs found on the bottom board to compare beetle presence before and after inspection across the study sites. We found that the percentage of SHBs found on the BB was significantly different between sites (Kruskal–Wallis test, *p* < 0.0001, H = 38.44, Figure 1b).

A post hoc Dunn’s test also showed that the percentage of beetles on the BB in CR and HI was not significantly different (*p* > 0.05). Mexico, however, had a statistically higher percentage of beetles on the bottom board before inspection compared to the other two sites (*p* < 0.001 for both HI-MX and CR-MX pairs). There were no statistically significant differences in beetle proportions on the bottom board after inspection across the three sites (Kruskal–Wallis test, *p* > 0.05, Figure 1c).

### 3.4. Inspection of the Cover and First Box

The small hive beetle has been reported to favor the edges of the bee nest and can sometimes be corralled in the periphery of the colony by worker bees [35]. Consequently, the top areas of the hive, including the lateral frames, could hold an important percentage of the beetle population. We present data on the percentage of beetles found on the cover, the side frames (Sf_T_), and the center frame (Cf_T_) of the top box (the one immediately under the cover).

#### 3.4.1. Cover Inspection

**Figure 2:**
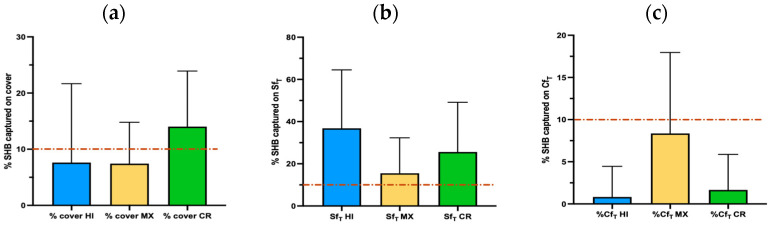
The average percentage of SHBs found on (**a**) the cover, (**b**) the topmost side frames (Sf_T_), and (**c**) the center frame of the topmost box (Cf_T_). The dotted line, which indicates a 10% prevalence, is included as a reference to assist in visually comparing the prevalence in different hive areas at the three study sites.

The SHB abundance on the cover was relatively low for all sites (Figure 2a). However, there was a significant difference between the sites (Kruskal–Wallis test, *p* < 0.05, H = 8.58). A post hoc Dunn’s test revealed that among the three pairs, only HI and CR were significantly different from each other (*p* < 0.01, Z = 2.87).

#### 3.4.2. Side Frame Inspection

We found that the prevalence of SHBs on the SF_T_ was higher than the percentage of SHBs on the cover for the three sites (Figure 2b). The percentage of beetles on the side frames was significantly different between the sites (Kruskal–Wallis, *p* < 0.05, H = 7.79). Based on the results of a post hoc Dunn’s test, we found that the only pair significantly different was HI vs. MX (*p* < 0.01, Z = 2.74).

#### 3.4.3. Center Frame Inspection

The center frame of the top box had a relatively low SHB prevalence (below 10%) at all sites (Figure 2c). However, there was a significant difference between the three sites (Kruskal–Wallis, *p* < 0.0001, H = 26.55). A post hoc Dunn’s test revealed that the average percentage of SHB caught in the top center frame in Hawai’i and Costa Rica was not statistically different (*p* > 0.05). In contrast, the average SHB percentage captured on the CF_T_ in MX significantly differed from HI and CR (*p* < 0.0001 for both pairs, Z values of 5.05 and 3.98, respectively).

### 3.5. Defense of the Brood Nest

From the perspective of a nest parasite such as the SHB, the brood nest is a highly desirable area because it contains important nutritional resources, including bee larvae, pupae, and pollen. At the same time, honeybees would be expected to invest more energy in defending this area, too. Bee broods were available in all the bottom brood boxes, and to standardize the comparison, we restricted our analysis to only single deep boxes at all sites. Consequently, we examined the prevalence of SHBs in the center frame in 10 colonies in HI, 14 in MX, and 7 in CR. The prevalence of SHBs in the Cf_b_ was only 0.13% of all beetles caught for HI; no beetles were caught in the C_b_ in Costa Rica, and in Mexico, an average of 8.4% of all beetles were caught in the brood nest. The difference in the percentage of SHBs captured in the Cf_b_ in single deeps was statistically significant between sites (Kruskal–Wallis test, *p* < 0.0001, H = 19.27). At the Mexico apiary, colonies with below-average SHB infestation had fewer beetles in the Cf_b_ compared to colonies with above-average overall beetle infestation, 2.5 versus 21 beetles/colony, respectively (Mann–Whitney U-test, *p* < 0.05, U = 7).

### 3.6. Summary: Overall SHB Distribution in the Colonies

**Figure 3:**
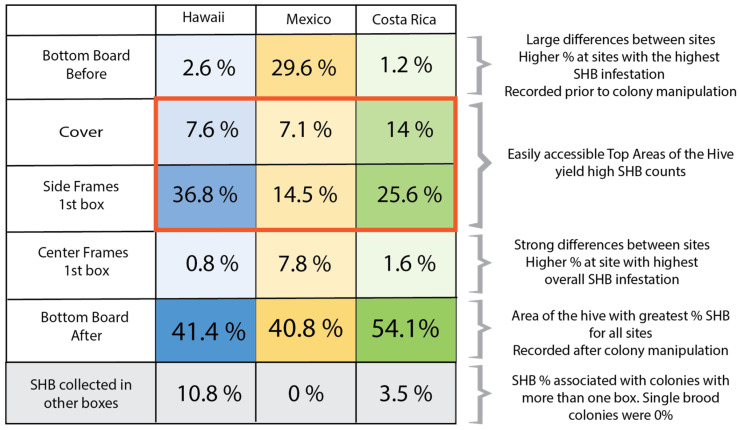
The proportional distribution of SHBs within the colonies. The intensity of the color blocks in each column reflects the proportion of SHBs found in each area. The blocks inside the red outline represent the average percentage of SHBs captured in the top areas of the hive. Gray-colored blocks represent SHBs that were captured within the hive but not on the bottom board, neither before nor after inspection.

There was significant variability in overall beetle density at our study sites. Nevertheless, we found some overarching patterns (Figure 3). First, the number of beetles on the bottom board before an inspection was lower than after the inspection was completed. Our field data also showed that the top areas of the hive, the cover, and the SF_T_ harbored a significant proportion of the beetles. Roughly 20 to 40% of all beetles caught came from those areas. The SHB density on center frames was significantly lower than that of the side frames for Hawai’i and Costa Rica, even when the SF % was divided by two to acknowledge that twice as many frames were sampled on the edge compared to a single frame in the middle. In Mexico, where the overall beetle population was high during that period of the study, there was a higher level of beetles on the center brood frame compared to the other two sites. The colonies sampled in Hawaii and Costa Rica included some colonies with multiple boxes compared to Mexico, where all the colonies sampled had just one deep. During the inspection of the taller hives, a percentage of the total beetles was captured as they moved downward following the start of the inspection but before they reached the bottom board of the colony. In Hawaii, where configurations included colonies with up to three supers, an additional 10.8% of SHBs were caught below the first box. In Costa Rica, where there were some super colonies, 3.5% of SHBs were caught on the boxes below the first box examined.

### 3.7. Targeted Sampling and Relative SHB Infestation Correlates

**Figure 4:**
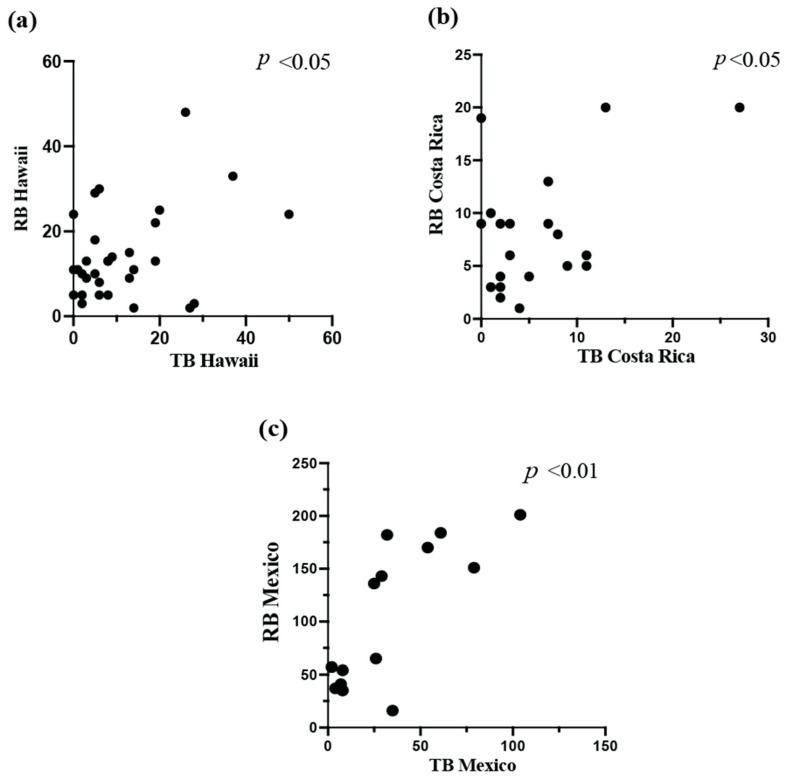
The results of the Pearson correlation analysis for the top areas of the hive (TBs) and the remaining beetles (RBs) across the three sites ((**a**) Hawaii; (**b**) Costa Rica; (**c**) Mexico). The *p*-values are listed at the top right of each graph. The Mexico data in (**c**) were calculated without an outlier point (see Section 3.8 below).

During a regular health inspection of a colony, the top box is easily accessible by beekeepers. Consequently, we examined whether the top areas of the hive could be used as a proxy for relative colony infestation. At each site, we tested how the number of beetles found on the cover, on the Sf_T_, and the sum of beetles caught in the cover and Sf_T_ (labeled as “top beetles”, TB) were correlated with the number of SHBs that were captured elsewhere in the hive (labeled remaining beetles, RBs) (Figure 4).

In Hawai’i, there was no correlation between the number of beetles caught on the cover or the Sf_T_ and the RBs. However, the sum of beetles from the top areas of the hive (TB) was positively correlated with the remaining beetles caught (Pearson correlation *p* < 0.05, r = 0.36, Figure 4a). In Costa Rica, there was a positive correlation between the three types of beetle counts and the RBs (Pearson correlation for all tests; cover *p* < 0.001 r = 0.72, Sf_T_ *p* < 0.05 r = 0.44, TB *p* < 0.05, r = 0.47, Figure 4b). In Mexico, there was a positive relationship between the number of beetles captured on the cover and the RBs (*p* < 0.05, r = 0.56), but not for Sf_T_. There was, however, a positive correlation between the total beetles caught on the top of the hive, TBs, and the remaining beetles, RBs (*p* < 0.01, r = 0.76, Figure 4c).

### 3.8. Notes on Effective Field Sampling of SHBs

**Figure 5:**
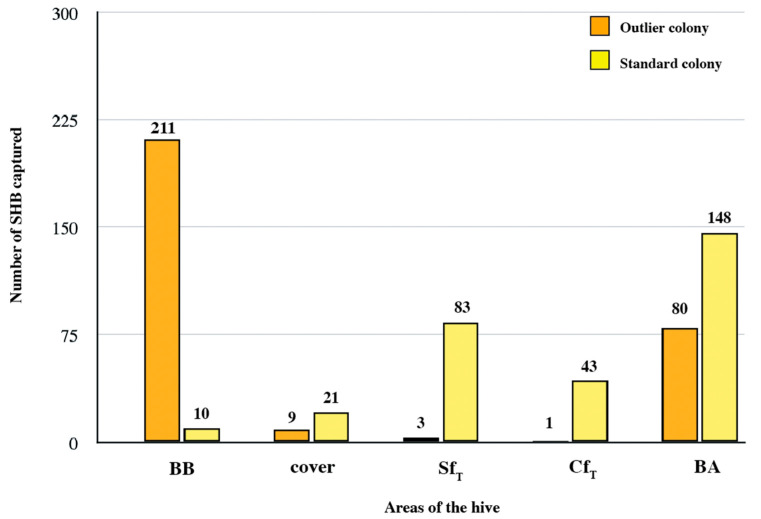
The comparative distribution of SHBs captured in different areas of the hive between the two highly infested colonies at the Mexican site. The areas of the hive include the bottom board before inspection (BB), the cover, the side frames of the top box (Sf_T_), the center frame (Cf_T_), and the bottom board after inspection (BA).

In this study, we had a colony in Mexico with a very high level of SHBs on the bottom board before inspection (211 compared to an average of 30 SHBs for the rest of the colonies at this site). We used the interquartile range (IQR = Q3 − Q1) to determine the upper boundary for the BB defined as “Q3 + 1.5∙QR”. The results suggest that the upper boundary for the BB is 104.5; consequently, the 211 SHBs captured on the BB firmly qualify as outliers. Since the outlier colony was heavily infested (304 SHB adults were captured), we selected a similarly highly infested colony (305 SHBs captured) at the same site to comparatively examine the in-hive beetle distribution of these two colonies (Figure 5). The results show that although these two colonies had the highest SHB infestation in the apiary, they exhibited a notably different in-hive SHB distribution (Figure 5). The proportion of adults on the BB was 69.4% for the outlier colony and 3.3% for the “normal” colony. The outlier colony had few adults in the Sf (0.9%) compared to the standard colony (27.2%). There were fewer SHBs in the Cf in the outlier colony than in the standard colony, 0.3% versus 14%, respectively. Potential explanations for the observed results are included in the discussion, along with comments on how hive components and colony management strategies may also impact in-hive distribution of the beetle.

## 4. Discussion

Our study provided novel data on the in-hive distribution of SHBs in naturally infested colonies of Africanized bees (*A. m. scutellata* × European racial hybrids) and honeybees of European ancestry. Using a modified visual inspection protocol, we confirmed that the majority of SHB adults did not reside on the bottom board. Instead, the floor of the hive became flooded, with beetles retreating downward to avoid disturbances as the inspection proceeded. Although the standard SHB visual screening [35] undoubtedly yields a highly accurate estimate of beetle infestation [47], the fast-paced selective screening we employed allowed us to gain a “snapshot” understanding of in-hive beetle distribution with less effort and provided a rough estimate of colony infestation. Furthermore, there was a positive correlation between the sum of beetles captured on the cover and side frames of the top box (TB) and the remaining beetles captured in the hive. As such, a beekeeper could examine easily accessible areas of the hive and gain knowledge about the relative infestation in a hive.

Small hive beetles quickly respond to the physical disturbances of the hive. Consequently, their distribution within beehives has proven challenging to examine. Nevertheless, a seminal paper by Neumann et al. (2016) [17] described the dynamics of behavioral interactions between bees and beetles in a natural cavity and indicated that there is an uneven distribution of beetles within the hive. The authors portrayed the beetle/bee conflict as a “tug of war” with attempted intrusions by the beetle to the brood nest and the bees’ diligently defending the brood area, but less so the honey frames. Our study found a much lower proportion of SHBs in the brood nest of EHB colonies than in the EHB colonies examined by Spiewok et al. (2007) [47]. Nevertheless, the proportion of SHBs that reached the brood nest in their artificially infested nuclear colonies was higher for EHBs than for African honeybees. These studies show that the defensive behavior of the bees influences the in-hive beetle distribution. Spiewok et al. [47] also confirmed that the in-hive distribution of SHBs was influenced by the presence and defensive behavior of worker bees. Nevertheless, the proportion of SHBs that reached the brood nest in their artificially infested nuclear colonies was higher for EHBs than for African honeybees. The difference in the brood infestation between the two subspecies of honeybees was hypothesized to be the result of either increased defensiveness on the part of African bees and/or the impact of hot and humid weather in Florida, which drove more bees out of the nest area. Our study confirmed that the proportion of SHBs found in the brood nest of EHB colonies in Hawaii was much lower than that found in the EHB colonies examined by Spiewok et al. (2007) [47]. A possible explanation for the observed discrepancy in SHB distribution could derive from the experimental setup of the Florida study with an artificial input of beetles to nuclear colonies versus observations of natural SHB infestations in larger colonies in Hawaii. Currently, there are insufficient data to refute or confirm the impact of ambient temperature and humidity on the distribution of bees or SHBs within the hive.

In this study, there were significant differences in the apiary infestation levels and in the proportion of SHBs captured in the brood nest between the two populations of Africanized bees. In Costa Rica, where overall SHB infestation was low, no SHBs were found in the brood frame. In the Mexico apiary, during the study period, we observed that the overall number of SHBs was much greater than at the other two sites, and the proportion of SHBs in the center frames was also relatively higher than Costa Rica and Hawaii. The impact of infestation levels on SHB distribution was obvious within the Mexico apiary, where colonies with high SHB populations also had more SHBs in the brood nest. Our findings suggest that the overall apiary infestation registered during certain periods of the year may be more significant than honeybee race in determining the success of brood nest defense.

The SHB is an agile insect that swiftly moves across the honeycomb and adopts protective stances when detected by bees [17]. Nevertheless, it has long been proposed that adult SHBs favor the bottom board, where they can feed on pollen pellets and other debris that falls from the brood chamber [48]. However, evidence indicates that honeybees construct prisons and encapsulate SHBs primarily on the nest’s periphery, rather than on the floor of the hive [49]. Our results showed that the proportion of SHBs on the bottom board before inspection was variable across sites and appeared to be positively linked to the overall apiary infestation. In contrast, as the colony was inspected, the proportion of beetles migrating downward was similar between sites. Thus, it is possible that when utilizing the standard visual inspection protocols, the accumulation of beetles on the floor of the hive has sometimes been interpreted as a biological preference for this area when it may have simply been induced by the physical disturbances involved in colony screening.

The historically reported high prevalence of adult *A. tumida* on the floor of the hive has impacted the focus of SHB control research, resulting in several studies focusing on insecticide treatments delivered in bottom-board traps [50,51,52]. The floor of the hive traps has been promoted as an easy, cheap, and less intrusive strategy to detect SHBs because the trap placement and removal does not require opening the hive. A study involving pesticide-baited traps positioned on the floor of the hive provided evidence that small hive beetles can take refuge under these traps and that the presence of a trap may lead to an increased number of SHBs on the bottom board [43]. However, more research is needed to ascertain whether the introduction of corrugated traps can enhance the attractiveness of bare bottom boards in colonies that naturally exhibit low SHB infestation levels, such as Hawai’i and Costa Rica. It is also unclear whether at low infestation levels enough beetles would enter the corrugated traps in 48 h to successfully assess the SHB infestation of colonies, as has been suggested [29].

The beekeeper’s equipment and management style may impact the SHB monitoring strategies’ success. The most recommended sampling methods for SHBs are based on visual inspection and in-hive traps [53]. The standard protocol for visual inspections is time-consuming and requires extensive manipulation of the colony, including lifting and shaking bees from individual frames [35]. This degree of manipulation would be difficult to achieve with full-sized colonies, especially when the bees are Africanized or during periods of dearth that can trigger robbing between colonies. To ensure quick access to different areas of the colony, the hive should be under regular maintenance; that is, no excess wax or propolis should be present to minimize the risk of hive components being stuck together during the screening. Excessive force and extensive manipulation needed to separate hive components could trigger the downward displacement of the beetles within the hive and allow beetles to fly away. In this study, it is possible that the outlier colony we observed in Mexico was inadvertently handled more forcefully than the other colonies, which could have resulted in an unusual distribution of SHBs in this colony compared to colonies with a similar level of SHB infestation.

The effective use of bottom-board traps can be impacted by uneven surfaces, which allow the beetle to scamper under the trap. In Latin America, where disadvantaged beekeepers build their own boxes, and occasionally use scrap materials for bottom board construction, the gaps and unevenness on the floor of the hive could interfere with corrugated traps that need to lay flat within the hive. In addition, sampling with in-hive traps, including bottom-board corrugated traps, requires repeated visits. An initial visit to set up the traps and a second one (within days) to retrieve the traps. Traveling to isolated apiaries in rural areas may be difficult and costly for beekeepers. This is especially true for low-income beekeepers in Latin America, where access and resources may be limited. Additional examples of how equipment and management may impact visual screening are presented in video format in Supplementary Materials.

A rapid screening of the top areas of the hive, assuming regular maintenance and normal levels of disturbance during the inspection, could help alert beekeepers if there has been a relative increase in beetle density from one visit to the next. If SHB levels increase, it is prudent to check the number of beetles in the center frames and assess the bee population. These simple steps could provide the beekeeper with an indication of whether there is a need to intervene and reduce adult beetle populations. It is important to note that during this study, there was no evidence of mass SHB reproduction in any of the hives, and larvae were not detected at any of the sites. This suggests that despite the differences in infestation levels, the individual colonies studied had sufficient bee coverage on the frames to prevent SHB reproduction. Nevertheless, persistent high densities of SHBs within colonies create a threat and the potential for a change in the bee vs. beetle dynamics that could result in colony loss or “slime out”. Monitoring relative infestation and noting the presence of SHBs in the nest area could allow beekeepers to determine when management strategies are needed at an apiary.

At a global level, the dispersion ability and the geographic range of invasive bee pests, including the SHB, are being impacted by climate change [54]. Recently, it has been shown that in Florida, where *A. tumida* has long been established, dispersal flights are positively linked to higher ambient temperatures [55]. Consequently, the predicted increase in temperatures across the globe could favor the spread of the SHB not only to higher latitudes but also across altitudinal gradients in montane tropical regions, where native bee diversity and the density of *Apis mellifera* may be different [56]. The potential geographical expansion of the SHB range will test the willingness of beekeepers to adopt new management strategies. A recent study by Vercelli et al. (2021) [57] showed that beekeepers’ opinions on climate change impacted their motivation to provide supplemental feeding and pest management needed to mitigate the climatic stressors affecting their colonies. In the case of the SHB, practical information about the beetle’s biology, in conjunction with easy-to-implement field assessments, will allow beekeepers to examine more colonies and make informed decisions in real time.

Finally, a cautionary note: although a flash screening of the cover and side frames appears to be useful to implement management decisions when the beetle is established, there is evidence that the levels of infestation during the initial spread of SHBs may be very low, and visual inspection alone may not be sufficient [46]. Thus, following the recommendations of establishing sentinel apiaries [4,32], preferably with in-hive traps armed with a killing agent (e.g., cooking oil), and conducting detailed visual inspections on those hives on a regular schedule remains a better option for detection.

## Figures and Tables

**Table 1:** Summary of the number of colonies sampled at each site and the corresponding hive configurations.

Sites	Total Number of Colonies Sampled	Colonies with a Single Brood Box	Colonies with Brood Box and Supers
HAWAII	31	10	21
MEXICO	14	14	0
COSTA RICA	20	7	13

## Data Availability

The authors confirm that the data supporting the findings of this study are available within the article.

## Supplementary Materials

The following supporting information can be downloaded at: https://www.mdpi.com/article/10.3390/insects16050517/s1.
